# Matrikines of Sea Cucumbers: Structure, Biological Activity and Mechanisms of Action

**DOI:** 10.3390/ijms252212068

**Published:** 2024-11-10

**Authors:** Aleksandr Popov, Emma Kozlovskaya, Tatyana Rutckova, Olga Styshova, Vyacheslav Makhankov, Aleksey Vakhrushev, Dmitry Hushpulian, Irina Gazaryan, Oksana Son, Ludmila Tekutyeva

**Affiliations:** 1 G.B. Elyakov Pacific Institute of Bioorganic Chemistry, Far Eastern Branch of the Russian Academy of Science, 159 Prospect 100-Letiya Vladivostoka, Vladivostok 690022, Russia; tanya1119@yandex.ru (T.R.); krivoshapkoon@mail.ru (O.S.); mvvslav@mail.ru (V.M.); aivahr@mail.ru (A.V.); 2 Faculty of Biology and Biotechnology, National Research University Higher School of Economics, 13/4 Myasnitskaya Str., Moscow 117997, Russia; hushpulian@gmail.com (D.H.); igazaryan@gmail.com (I.G.); 3 Bach Institute of Biochemistry, Federal Research Centre “Fundamentals of Biotechnology” of the Russian Academy of Sciences, Leninski Prospect 33, Moscow 1190721, Russia; 4 Department of Chemical Enzymology, M.V. Lomonosov Moscow State University, Moscow 119991, Russia; 5 Department of Chemistry and Physical Sciences, Dyson College of Art and Sciences, Pace University, 861 Bedford Road, Pleasantville, NY 10570, USA; 6 Department of Bioeconomy and Food Security, School of Economics and Management, Far Eastern Federal University, Vladivostok 690922, Russia; oksana_son@bk.ru (O.S.); lat7777@mail.ru (L.T.); 7 ARNIKA, Territory of PDA Nadezhdinskaya, Primorsky Krai, Volno-Nadezhdinskoye 692481, Russia

**Keywords:** matrikines (MKs), trepang *Apostichopus japonicus* MKs (MKT), biological activity, sea cucumbers, enzymatic transformation

## Abstract

Matrikines (MKs), the products of enzymatic fragmentation of various extracellular matrix (ECM) proteins, regulate cellular activity by interacting with specific receptors. MKs affect cell growth, proliferation, and migration, can induce apoptosis and autophagy, and are also effectively used in biomedicine and functional nutrition. Recently, there has been great interest in the structural features and biological activity of MKs from various sources. This review summarized and analyzed the results of modern research on MKs from sea cucumbers, primarily from trepang (MKT). Particular attention is paid to the analysis of the existing knowledge on the antioxidant, anti-inflammatory and adaptogenic activities of these MKs and the possible mechanisms of their protective action.

## 1. Introduction

In recent decades, various types of echinoderms have gained popularity among researchers not only because of their nutritional value but also because of their potential health benefits as important elements of functional nutrition, as well as for medico-biological and cosmetic use [1,2]. According to ancient manuscripts of the Ming Dynasty (1368–1644 BC), the Japanese sea cucumber (trepang) *Apostichopus japonicus* (*Stichopus japonicus*) possessed medicinal properties similar to ginseng; hence, it was also called “haishen”, meaning “sea ginseng”. Since ancient China, it has been believed that regular consumption of trepang rejuvenates and invigorates the human body [3]. Recent studies of its composition have shown that trepang, as a delicacy, is indeed a treasury of unique metabolites, trace elements, vitamins and other bioactive compounds essential for human health [1,4,5]. Matrikines (MKs) of trepang, as well as other commercially valuable sea cucumbers, are formed as a result of fragmentation of various extracellular matrix (ECM) components. These MKs are a valuable source of bioactive peptides that support human health and lower the risk of chronic diseases [1,2,6].

**Figure 1:**
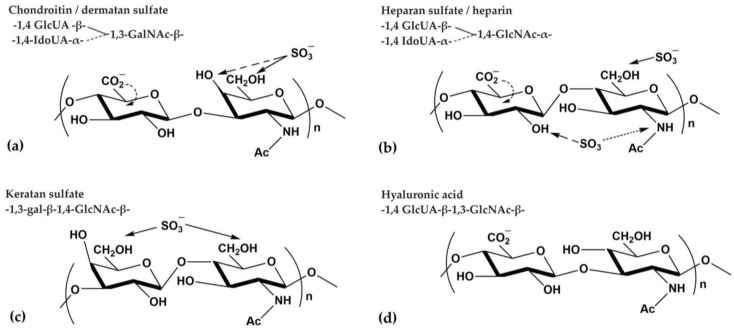
Various types of glycosaminoglycans (GAG) carbohydrate monomers: (**a**) chondroitin/dermatan sulfate, (**b**) heparan sulfate/heparin, (**c**) keratan sulfate, (**d**) hyaluronic acid. Abbreviations: GlcUA, glucuronic acid; GlcNAc, N-acetylglucosamine; IdoUA, iduronic acid; GalNAc, N-acetylgalactosamine; gal, galactose.

Presently, MKs of sea cucumbers have become the subject of research not only as important elements of functional nutrition but also for their effective use for medico-biological purposes. This review presents an analysis of recent literature regarding the structure, antioxidant, anti-inflammatory, and adaptogenic properties of MKs derived from sea cucumbers. The review also explores the potential mechanisms of MKs protective action, highlighting possible prospects for the development of new therapeutic-preventive agents, as well as functional nutrition components. It is important to note that the review is devoted to the analysis of data from more than 100 published articles, mainly after 2020, on antioxidant, anti-inflammatory and adaptogenic activities from sea cucumbers (MKs), mainly Far Eastern trepang (MKT). References are presented in such databases as Scopus, ResearchGate, PubMed, Google Scholar, and others. The results of the analysis and generalization of selected experimental studies are based on more than 50 different literature sources, which present experimental data indicating the high protective adaptogenic activity of these matrikines and the methods of their adaptogenic action in the form of one table and six figures. The review also provides data on carbohydrate residues included in the structure of MKs and MKT (Figure 1).

## 2. General Characteristics of Structure and Properties of Matrikines

The structure–function relationship of MKs is actively studied for its use in functional nutrition, tissue engineering, and regenerative medicine [7]. The primary advantage of regulatory MKs over chemical drugs is that, as analogs of endogenous compounds, they typically result in fewer side effects and often demonstrate a significant therapeutic effect at relatively low doses.

The activity of these molecules is determined by their unique amino acid sequences and compositions. The size of the peptides can range from three to twenty amino acid residues. Because they are produced naturally in the body, their small size and low species specificity, along with the presence of highly specific receptors on cell membranes, allow MKs to display multifunctional characteristics. This makes them potentially valuable for therapeutic applications and dietary use [7,8].

Sea cucumbers contain nutritional and biologically active components, such as ECM proteins, collagens, and proteoglycans (the main source of specific MKs in sea cucumbers), as well as saponins, oxygenated carotenoids and sulfated polysaccharides, primarily fucoidan sulfate (FS) and fucosylated chondroitin sulfate, possessing a wide range of medical-biological and functional activities [9].

Proteoglycan fragments of MKs from sea cucumbers are glycoconjugates consisting of a protein “core” to which three types of carbohydrate chains can be covalently attached. These three types of carbohydrates include sulfonated glucosamine residues (GlcNSO_3_), N-acetylglucosamine (GlcNAc), or N-acetylgalactosamine (GalNAc), alternating in glycosidic bonds with glucuronic acid (GlcUA), iduronic acid (IdoUA), or galactose residues. Distinct patterns of disaccharide repetitions result in the creation of different types of unbranched glycosaminoglycans (GAGs), which typically range in size from 20 to 40 kDa. The various types of GAGs include chondroitin and chondroitin sulfate (CS), dermatan and dermatan sulfate (DS), heparin and heparan sulfate (HS), as well as keratan and keratan sulfate (KS) (refer to Figure 1a–c). These GAGs are variably substituted with sulfate linked to a free amino group and/or hydroxyl group [10,11].

GAGs are covalently attached to the core protein via an O-glycosidic bond formed with the amino acid serine. Of note, hyaluronic acid (HA) is not covalently bound to the protein, so it is not proteoglycan-forming (Figure 1d). Typically, one type of GAG predominates in a given core protein, but hybrid proteoglycans also exist. All GAGs carry a negative charge due to sulfate and carboxyl groups, with heparin being the most sulfated compound found in living tissues. The presence of a high negative charge distinguishes proteoglycans from other glycoconjugates [10,11].

Sea cucumber’s proteoglycans are primarily composed of fucosylated chondroitin sulfates (FCSs) and sulfated fucans (SF). FCS is a unique ingredient found specifically in sea cucumbers. FCSs are branched glycosaminoglycans (GAGs) that features a backbone similar to chondroitin sulfate (CS), consisting of repeating disaccharide units made up of N-acetylgalactosamine (GalNAc) and glucuronic acid (GlcA) residues. Unlike the linear structure seen in mammalian CS, FCSs feature fucosylation on the GlcA and/or GalNAc residues, which gives them a branched structure. Various factors influence the biological activity of FCS, including the placement of sulfate groups, the level of sulfation in both the main chain and its branches, as well as the number and arrangement of fucosyl branches [10,11].

One of the important advantages of sea cucumbers is their unique texture, which is soft and quite elastic. Several studies report changes in the texture of sea cucumbers during processing, such as boiling and resorption [2,8]. Additionally, sea cucumbers are prone to autolysis and subsequent texture deterioration, which is supposed to be catalyzed by certain endogenous enzymes, such as cysteine proteinases and metalloproteinases. Previous studies have shown that the body wall of the sea cucumber mainly consists of mutable collagenous tissue, which is an excellent example of biological materials capable of rapidly changing their stiffness and extensibility under the control of the nervous system [12].

A distinctive feature of sea cucumbers is their remarkable regenerative ability. If the body of a holothurian is torn into three parts, within 4-7 months, three new individuals will regenerate. Their unique property of making their body rigid when threatened and then returning to a gelatinous state is also notable due to the presence of mutable collagenous tissue (MCT) [2,6].

The proteolytic cleavage of the ECM by matrix metalloproteinases (MMPs) and/or conformational changes unmask “cryptic” sites and release fragments with biological activity that is not observed in the intact molecule. The identification of bioactive cryptic regions in the MCT of holothurians and other echinoderm species attracts the attention of researchers worldwide, as these bioactive peptides can be used in the development of biomaterials and tissue engineering [2,13].

The body walls of the sea cucumber *A. japonicus* mainly consist of collagens and proteoglycans, which account for approximately 70% of the total protein [14,15]. These diverse protein components of the ECM of sea cucumbers are a rich resource for the preparation of bioactive peptides, in particular MKT, with regulatory properties and anti-inflammatory effects [2,16,17,18].

Thus, MKTs play a significant but insufficiently studied role in chronic and age-related diseases, as well as in fatigue syndrome caused by increased physical and mental stress. Understanding interstitial communication is of particular interest. Knowledge about MKTs and their role in controlling the pathology processes of internal organs can be used to develop effective innovative treatment methods [2,19].

Researchers are particularly interested in the peptides of MKs and MKT, which have a positive corrective effect on the functions of the body in experimental modeling of various pathologies and may be beneficial for human health [8,20,21].

## 3. Antioxidant and Anti-Inflammatory Activity of MKs and MKT

Excessive production of free radicals is an important cause of oxidative stress. Oxidative stress, prolonged work and high-intensity exercise can lead to excessive free radical loads, which easily lead to the development of a wide range of inflammatory diseases [2,4]. A wide range of recent studies of MKs and MKT obtained by enzymatic hydrolysis of sea cucumber have attracted the attention of specialists with the prospect of using these marine peptides for the treatment or prevention of chronic diseases, considering their antioxidant and anti-inflammatory potential, as well as their high commercial value [2,21,22,23].

The reported molecular weight of MKT obtained as a result of trypsin hydrolysis varied from 5 to 25 kDa. These peptides were rich in glycine, alanine, glutamate, proline and hydroxyproline residues and demonstrated high efficiency in scavenging radicals [24]. Two tetrapeptides (Val-Thr-Pro-Tyr and Val-Leu-Leu-Tyr) and a hexapeptide (Val-Gly-Thr-Val-Glu-Met) from MKT exhibited protective effects against DNA damage caused by toxic hydroxyl radicals. MKT peptides with a molecular weight of less than 3 kDa have the ability to relieve fatigue caused by heavy physical activity, probably due to the normalization of energy metabolism. They protect cells and tissues of the body from oxidative damage by modulating the synthesis of cytokines and reducing the overexpression of toll-like receptors and the nuclear factor kappa-light-chain-enhancer of activated B cells (NF-κB) [25].

The anti-inflammatory properties of MKs of various origins have been known for many years. Recently, significant progress has been made in understanding the mechanism of antioxidant and anti-inflammatory activity of a number of MKs: it has been shown that they have the ability to specifically bind to ECM glycoproteins and/or adsorb on their surface and thereby change the conformation of receptor protein molecules and influence their signaling properties. Such mechanisms play an important role in matrix remodeling in response to acute inflammation and/or oxidative stress [2,26].

A common approach to assessing the acute anti-inflammatory activity of compounds in in vivo animal models is to test their ability to reduce or prevent the development of carrageenan-induced edema. CD-1 mice (20 ± 2 g) were used as a test system to evaluate the anti-inflammatory properties of MKs marine echinoderm in comparison with nonsteroidal anti-inflammatory drugs (NSAIDs), which inhibit the production of prostaglandins. Five experimental samples of marine echinoderm MKs were studied: sea cucumber *A. japonicus* (MKT), cucumber *Cucumaria japonica* (MKC), sea urchins *Scaphechinus mirabilis* (MKUm) and *Strongylocentrotus nudus* (MKUn) and sea star *Patiria pectinifera* (MKS). These were compared with the NSAID indomethacin [2,8]. When administered in doses of 10 and 20 mg/kg, MKUn exhibits high anti-inflammatory activity (Table 1). They reliably inhibit the formation of paw edema to varying degrees in the range from 34 to 56%, whereas indomethacin (at a dose of 10 mg/kg) inhibits it by 52.0 ± 4.06% compared to the control (group without treatment). In some cases, a dose of 20 mg/kg leads to a weaker protective effect than a dose of 10 mg/kg, which indicates the need for further study of the anti-inflammatory potential of MKs of different origins using lower therapeutic doses [8,21].

The antioxidant and anti-inflammatory activity of MKT and MKs of other sea cucumbers obtained by enzymatic hydrolysis has been studied by various research groups, including in vitro experiments [27]. When testing the anti-inflammatory effects of MKTs on RAW264.7 mouse macrophages stimulated with lipopolysaccharide (LPS), a major component of the outer membrane of Gram-negative bacterial cells, it was shown that they significantly reduced LPS-induced nitric oxide and mRNA synthase release without affecting cell viability. The mRNA expression of LPS-induced inflammatory cytokines, including tumor necrosis factor-α (TNF-α), interleukins (IL) IL-1β, and IL-6, was suppressed by MKT. They inhibited LPS-induced degradation of IκBα, (κBα inhibitor) and nuclear translocation of the NF-κB p65 signaling protein, which led to a decrease in trans-activation of NF-κB, a universal transcription factor controlling the expression of immune response genes, apoptosis and cell cycles [27]. Dysregulation of NF-κB causes a sharp increase in inflammatory processes, as well as the development of autoimmune lesions, viruses and tumor diseases. MKT suppressed LPS-induced phosphorylation of key signaling proteins JNK, ERK and p38. Moreover, under the influence of MKT, the expression of heme oxygenase-1 (HO-1) in macrophages increased in a dose-dependent manner. The anti-inflammatory effect of MKT, achieved by inhibiting the mRNA expression of pro-inflammatory cytokines induced by LPS, was partially reversed by the simultaneous administration of an HO-1 inhibitor [27].

Thus, MKTs have a pronounced anti-inflammatory effect, inhibiting the activation of the pro-inflammatory pathways NF-κB and mitogen-activated protein kinase (MAPK) and inducing the expression of antioxidant defense enzymes, which play a key role in restoring redox balance and physiological homeostasis. It is interesting that the most active MKTs were rich in glycine, glutamic and aspartic acids, and their molecular weights were predominantly <1000 Da (87.20%), indicating a high content of low molecular weight peptides consisting of 2-6 amino acid residues [27]. It was previously noted that dipeptides and tripeptides also effectively inhibit the inflammatory response of LPS-activated macrophages [28,29].

In other studies, a comprehensive assessment of the antioxidant activity of MKT was carried out using Vero cells and zebrafish—classic models for this type of testing—in vitro and in vivo, respectively. MKTs were obtained by treating the sea cucumber body with *S. japonicus* using various food enzymes (Alcalase^®^, α-chymotrypsin, Neutrase^®^, papain, pepsin, Protamex^®^ and trypsin). Antioxidant free radical scavenging activity in cells was determined using electron spin resonance spectroscopy. MKT obtained after treatment with chymotrypsin (α-chy) showed the highest activity in binding hydroxyl radicals. α-Chy had a high protein content and relatively low levels of polysaccharides and sulfates. They were subsequently fractionated by ultrafiltration according to molecular weight: >10 kDa, 5–10 kDa and <5 kDa (α-chy-III) and assessed for their antioxidant properties on Vero cells under H_2_O_2_ exposure. MKT from the α-chy-III fraction significantly reduced the production of intracellular reactive oxygen species (ROS) and increased cell viability. Notably, these MKTs were most effective in reducing intracellular ROS levels and increasing cell viability, as well as providing protection against H_2_O_2_-induced apoptotic damage. Interestingly, MKT from the α-chy-III fraction also significantly reduced the mortality of zebrafish embryos exposed to H_2_O_2_ and reduced lipid peroxidation [25,26].

In general, the results of the studies showed that α-chy hydrolysate and its α-chy-III fraction from *S. japonicus* have high antioxidant activity and can be used as active ingredients in functional nutrition. Studies of the antioxidant activity of MKT on Vero cells also showed that with increasing concentration, these peptides have a pronounced ability to neutralize free radicals and, as a result, a high potential for protection against oxidative stress [30,31]. The antioxidant and anti-inflammatory activities of these peptides have been confirmed in in vitro (cell culture) or in vivo animal models [25,30], but clinical testing in humans has not yet been performed.

Overall, the studies showed that the α-chy hydrolysate and its α-chy-III fraction from *S. japonicus* have high antioxidant activity and can be used as active ingredients in functional foods. In vitro studies of the antioxidant activity of MKT at the cellular level also showed that, with increasing concentration, these peptides have a pronounced ability to scavenge free radicals and, consequently, a high potential for protection against oxidative stress.

## 4. Structural Features of MKs on Affecting Their Antioxidant and Anti-Inflammatory Activity

The studies also indicate that positively charged residues are involved in the anti-inflammatory response of MKs [27]. Since the positively charged region of the peptide may behave as a chemokine, the peptides affect the immune response as a result of MKs binding to the corresponding chemokine receptors. Two basic amino acids (Arg and Lys) with a net positive charge at physiological pH at the N-terminal end are a general requirement for the high antioxidant and anti-inflammatory activity of MKs [28]. Cationic is a characteristic that most active peptides exhibit at physiological pH. However, terminal Arg on many of the more common MKs prevents inflammation since they adhere to polysaccharides secreted by bacteria and macrophages. It may indicate that polar amino acids at the C-terminus (e.g., lysine or histidine), which is a characteristic feature of anti-inflammatory peptides, could be a contributor to their anti-inflammatory response [25,27,30].

According to the published data, MK’s peptide chain length and the presence of a carbohydrate residue play an important role in biological activity [8,30]. Out of the 31 peptides studied, 6 are di- or tripeptides, while 19 contain 4–20 amino acids, suggesting that shorter amino acid sequences may have enhanced biological activity. Approximately 42% of the most biologically active peptides have a molecular weight under 1000 Da, with the rest not surpassing 3 kDa. Although a definitive link between proline, glycine, or glutamine in the peptide sequence and anti-inflammatory activity is yet to be confirmed, several shared characteristics seem to contribute significantly to their inflammatory response. These include the presence of hydrophobic amino acids (Val, Ile, Pro), positively charged amino acids (His, Arg, Lys), and carbohydrate residues. Other studies support the beneficial impact on anti-inflammatory activity from the presence of aromatic amino acid residues or proline at the terminal position. The combination of MK shows a greater anti-inflammatory effect than the individual MK peptides, suggesting potential synergism within the MK group [25,32].

It should be emphasized that further comprehensive studies, including clinical ones, are needed to clarify the stability of UA and MCT in the gastrointestinal tract, their bioavailability and interaction with food and the microbiome, and the safety of their use as potential preventive and treatment options for chronic inflammatory diseases. Furthermore, identifying these peptide sequences obtained through enzymatic hydrolysis will aid in the design and production of new anti-inflammatory peptides.

## 5. Plausible Pathways Leading to Protective Activity of MKs and MKT

### 5.1. Protective Effect of MKT Against High Physical Loads and Oxidative Stress

Exercise disrupts the energy balance in skeletal muscle and activates integrated cellular signaling networks to address the increased energy and oxygen demands that accompany muscle contraction. Research into exercise-induced changes in skeletal muscle signaling networks has identified new mechanisms through which exercise enhances mitochondrial biogenesis in skeletal muscle, contributing to overall health and fitness. While acute exercise influences a complex array of protein post-translational modifications (such as phosphorylation) in skeletal muscle, prior studies focusing on exercise signaling in human and rodent skeletal muscle have mainly targeted a limited selection of exercise-regulated protein kinases (like AMPK and MAPK) and a small number of their protein substrates. However, global mass spectrometry-based phosphoproteomic analyses have uncovered the extensive complexity and interconnectivity of exercise signaling pathways and kinases beyond this limited group, including the phosphorylation and/or translocation of various exercise-regulated mitochondrial and nuclear protein substrates [33].

Studies of the mechanisms of protective activity of MKs and MKTs were carried out both in vitro and in vivo. At the same time, a clear dose-dependent increase in the transcriptional activity and expression levels of nuclear factors PGC-1α, NF-κB and NRF2, which are key regulators of oxidative stress, with enhanced stabilization of their nuclear and mitochondrial translocation, was noted. Additionally, it has been found that therapeutic administration of MKT reduces intracellular levels of ROS while simultaneously increasing cellular resistance to H_2_O_2_. These findings lay the groundwork for further research into the antioxidant and anti-inflammatory effects of MKT. AMPK is required to increase peroxisome proliferator-activated receptor γ-coactivator-1α (PGC-1α) expression in skeletal muscle in response to creatine deficiency. PGC-1α is a transcriptional regulator of genes involved in fatty acid oxidation, gluconeogenesis, and is considered a master regulator of mitochondrial biogenesis [25,32,33,34]. It can be assumed that the protective effect of MKT under the conditions of oxidative stress is mediated through the AMPK signaling pathway and associated downstream factors.

**Figure 2:**
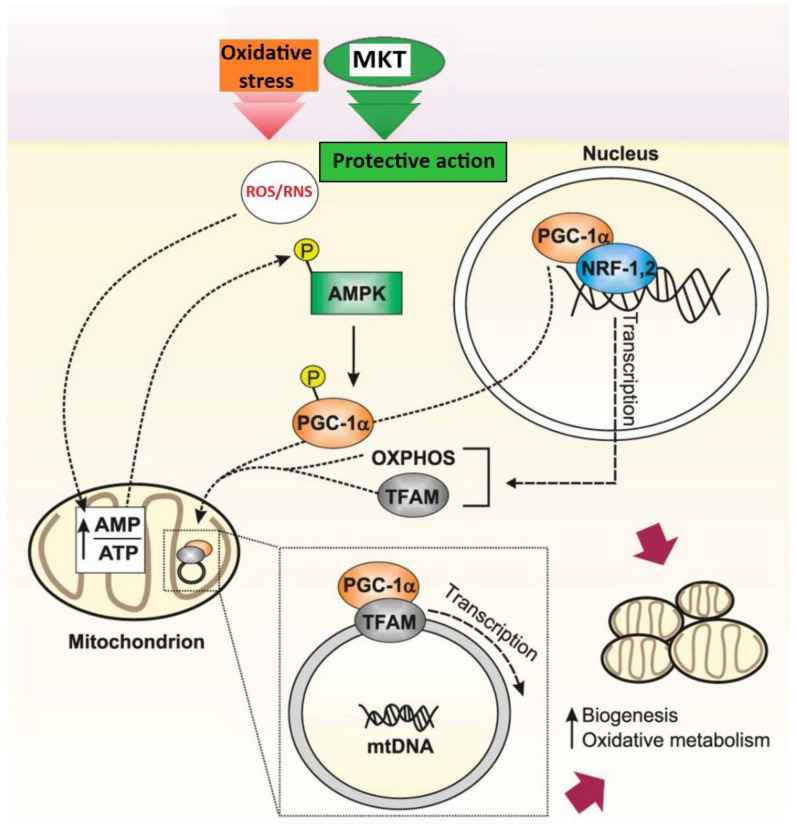
Proposed protective effect of MKT under the conditions of oxidative stress through the AMPK signaling pathway and associated downstream factors. Adapted from Ref. [35]. Abbreviations: ROS/RNS, reactive oxygen and nitrogen species; AMP, 5′ adenosine monophosphate; ATP, 5′ adenosine triphosphate; AMPK, 5′ adenosine monophosphate-activated protein kinase; MKT, matrikines *A. japonicus*; mtDNA, mitochondrial DNA; NRF-1,2, nuclear transcription factors (NRF-1, NRF-2); OXPHOS, oxidative phosphorylation; PGC, 1α-peroxisome proliferator-activated receptor gamma coactivator 1-alpha; TFAM, mitochondrial transcription factor A.

Figure 2 depicts the scheme for the regulation of redox processes and cellular metabolism through a signaling pathway with the main element being AMPK. Its activation occurs when the cell energy level is low, hence, all energy-consuming pathways like protein synthesis are inhibited, whereas energy-producing pathways are activated to restore the cellular energy level. AMPK influences antioxidant defense and stimulates mitochondrial biogenesis by regulating PGC-1α, which in turn promotes gene transcription in mitochondria. This pathway is activated in response to a significant cellular energy consumption (for example, during physical exercise) and an increase in intracellular levels of AMP. As a result of AMPK activation, the cell transitions into an energy-saving mode (including blocking the synthesis of fatty acids and activating their oxidation) [20,25].

It is important to note the AMPK pathway is one of the universal signaling pathways characteristic of most cells, associated with maintaining redox balance, eliminating signs of oxidative stress, and influencing numerous important functions [25]. The PGC-1α protein acts as a transcription coactivator that regulates genes involved in energy metabolism, the primary regulator of mitochondrial biogenesis, and biochemical processes associated with active skeletal muscle function. Its activity undergoes significant changes during physical work and training. PGC-1α activates two key transcription factors, namely NRF1 and NRF2. Upon activation, these transcription factors bind to DNA and initiate the expression of various proteins related to antioxidant response and mitochondrial biogenesis [32].

These proteins are then imported into the mitochondria, stimulating their growth and division. Mitochondria also possess their own mitochondrial DNA (mtDNA), containing 37 genes encoding proteins involved in respiration. In addition to activating genes in nuclear DNA, PGC-1α can also activate genes in mtDNA. For instance, PGC-1α activates another transcription factor called Tfam (mitochondrial transcription factor A). This activation involves the expression of mitochondrial genes associated with mitochondrial biogenesis and other adaptations to physical exertion [34].

There is clear evidence indicating that the phosphorylation and/or translocation of mitochondrial and nuclear proteins play a crucial role in the adaptive responses of skeletal muscle following an acute session of endurance exercise. This process ultimately promotes mitochondrial biogenesis and enhances the overall health and fitness benefits associated with exercise. In turn, MKT, recognized as an effective antioxidant, exhibits anti-inflammatory and adaptogenic activities by mitigating oxidative stress in experimental animal models.

### 5.2. Other Protective Mechanisms of MKs and MKT Against Inflammation and Fatigue

Physical exercise can lead to an excessive production of free radicals and disruption of cellular metabolism, which may result in fatigue. One significant mechanism of cellular damage caused by free radicals is lipid peroxidation, which can directly harm cell membranes and is linked to various pathological processes. Malondialdehyde (MDA), a byproduct of lipid peroxidation that occurs when reactive oxygen species (ROS) attack the body, serves as an indirect indicator of cellular damage. Research indicates that following MKT treatment, the levels of MDA in the serum and brains of mice decreased compared to the control group. Concurrently, the levels of essential antioxidant defense enzymes—including catalase (CAT), glutathione peroxidase (GPX), and superoxide dismutase (SOD)—increased significantly in both the serum and brains of the mice. These antioxidant enzymes are critical for suppressing ROS production and regulating energy and redox metabolism at the cellular level, thereby mitigating the onset of pathological conditions [32]. Thus, MKT demonstrates effective antioxidant properties by inhibiting lipid peroxidation and displaying anti-inflammatory and adaptogenic effects through the reduction in oxidative stress in experimental animal models.

**Figure 3:**
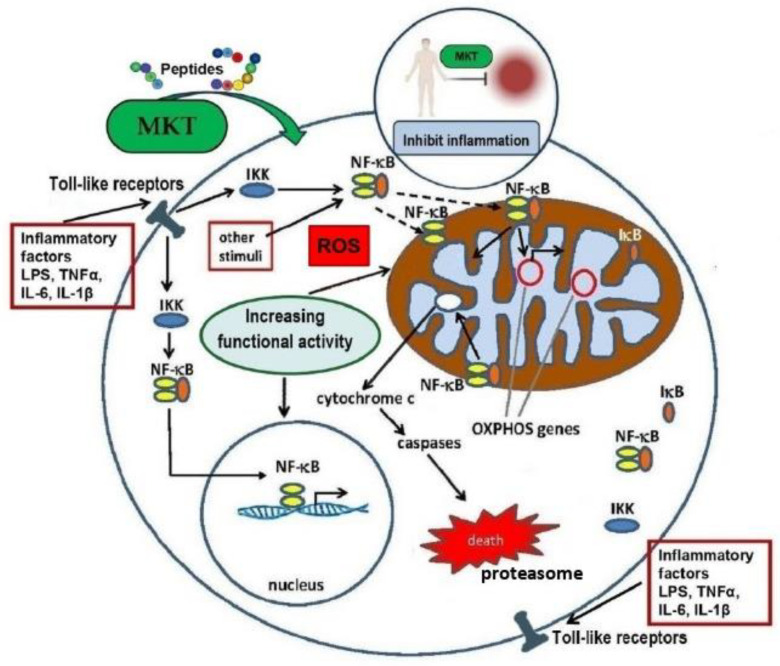
Possible mechanism of the MKT protective effect on inflammatory processes induced by ROS and inflammatory factors (LPS, NF-κB, TNFα, IL-6 and IL-1β). Adapted from Ref. [39]. Abbreviations: LPS, lipopolysaccharide; NF-κB, nuclear factor kappa-light-chain-enhancer of activated B cells; IκB, cytosolic inhibitory protein; IKK, IkappaB kinase; IL, 1β-interleukin-1beta; IL-6, interleukin-6; MKT, matrikines *A. japonicus*; OXPHOS, oxidative phosphorylation; TNFα, tumor necrosis factor.

Experimental data suggest that MKT may protect cells from excessive inflammatory responses by acting through the signal pathway NF-κB. As shown in Figure 3, NF-κB is activated by a variety of stimuli, including cytokines and stress factors such as reactive oxygen and nitrogen species (ROS/RNS). In the cell cytoplasm, NF-κB is in an inactive state in a complex with the inhibitory protein IκB. A stimulating agent activates the NF-κB signaling pathway, where IκB is phosphorylated by the IKK kinase (IκB kinase), leading to the degradation of IκB by the 26S proteasome. As a result, NF-κB is released from the inhibitory complex, translocates to the nucleus and activates the transcription of controlled genes [36,37,38].

The immune system’s ability to recognize MKs from sea cucumbers suggests that they may act as damage-associated molecular patterns (DAMPs), meaning they can initiate a non-infectious inflammatory response [37]. MKs have the function of releasing pro- and anti-inflammatory molecules such as interleukin 1 beta (IL-1β), tumor necrosis factor α (TNF-α), and cytokines (IL-6). Many of the pro-inflammatory effects of low molecular weight MKs are explained by their interaction with and activation of toll-like receptors [36,37,38].

Thus, judging by the results of therapeutic application, MKs lead to the activation of the NF-κB transcription factor, promoting its translocation to the nucleus and mitochondrion and the subsequent antioxidant and anti-inflammatory responses [31]. The effect of sea cucumber MKs is closely associated with signaling pathways that facilitate the removal of free radicals and enhance the molecular and cellular mechanisms of the body’s antioxidant and anti-inflammatory defenses [34]. Testing MKT using a swimming load model allowed for the assessment of its impact on the antioxidant potential of experimental animals. The ability of rats to withstand oxidative stress was significantly improved by oral administration of MKT [25]. Changes in the content of energy and metabolic markers, an increase in the levels of antioxidant defense enzymes, and biomarkers of oxidative stress were recorded. Additionally, MKT administration can modulate changes in inflammatory cytokines and suppress the excessive expression of pro-inflammatory factors via the NRF2 pathway. Of note, MKT mainly consisted of low molecular weight peptides (<2 kDa). Amino acid composition analysis showed that MKT was rich in glycine, glutamic acid, and proline [25,40]. The authors suggest that MKT has an anti-inflammatory effect likely due to the normalization of cell energy metabolism, as well as the reduction in oxidative damage and inflammatory responses, controlled through the NRF2 pathway.

Fatigue resulting from physical exertion prevents the body from sustaining a certain level of exercise intensity. One effective way to assess the physical endurance of animals is through a loaded swimming test. This model is particularly useful for examining the impact of treatments aimed at reducing oxidative stress [36]. The duration of swimming under load serves as a direct indicator of an animal’s physical endurance. Another common method for evaluating fatigue resistance is the pole-climbing test. In this assessment, mice are positioned head-up at the top of a pole, and they eventually slide off when they reach fatigue. Researchers measure and record both the time taken to turn around and the climbing duration. Notably, in this study, mice receiving both low and high doses of MKs outperformed those in the negative control group [36]. Mental fatigue, often stemming from inadequate rest or cognitive overexertion, is a challenge that affects many individuals, including adolescents and the elderly. To gauge changes in learning ability, mental state, and activity levels in animals, researchers utilize tests such as the passive avoidance test and the elevated plus-maze test. Findings indicate that animals treated with therapeutic doses of MKT demonstrated improved mental states and enhanced learning capabilities, which contributed to decreased mental fatigue. The results from these behavioral assessments suggest that MKT not only aids in recovery post-exercise but also alleviates cognitive fatigue in BALB/c mice [37].

It has been found that treatment with MKT peptides with a molecular weight of <5 kDa significantly increases the levels of energy-sensitive factors, lipid metabolism, as well as mRNA levels and protein factors of mitochondrial biogenesis in experimental animals. In addition, the levels of gluconeogenic mRNA are significantly elevated. This adaptogenic effect against fatigue can be explained by the improvement in mitochondrial function through energy regulation and enhanced antioxidant activity [25,37].

**Figure 4:**
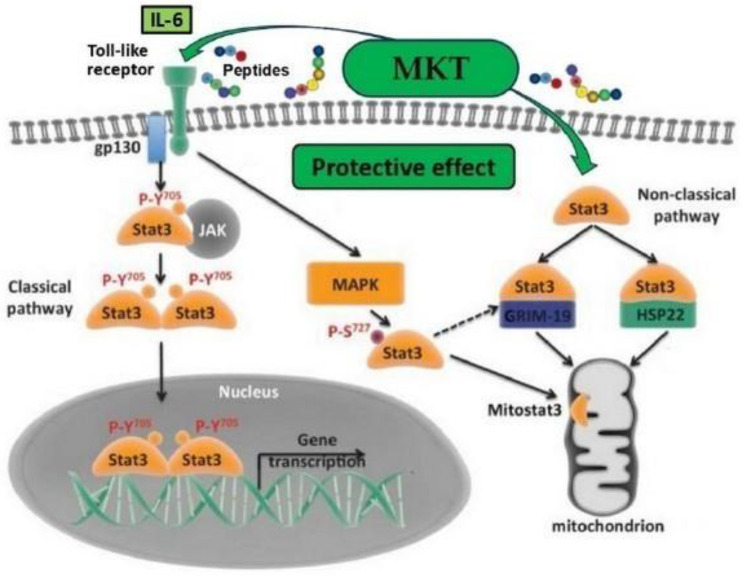
MKT may affect classical and non-classical pathways of STAT3 signaling. Adapted from Ref. [42]. Abbreviations: IL-6, interleukin 6; gp130, glycoprotein 130, expressed signal transducer; STAT3, transducer and activator of transcription family 3; JAK, Janus kinase; MAPK, mitogen-activated protein kinase; GRIM-19, a cell death regulatory protein; HSP22, heat shock protein; MKT, matrikines *A. japonicus*; Mitostat3, a small pool of Stat3 was found localized in mitochondria.

As shown in Figure 4, MKT with a molecular weight of <2 kDa, obtained using neutral protease, exhibited mechanisms of action that included reducing damage from oxidative stress and inflammation, regulating energy metabolism, and expressing toll-like receptors and NF-κB. NF-κB is activated by a variety of stimuli, including cytokines (such as TNF and interleukin 1), T- and B-cell mitogens, bacterial and viral products (all ligands of toll-like receptors, for example, lipopolysaccharide or double-stranded viral RNA), and stress factors (such as reactive oxygen species or ultraviolet light). NF-κB is a key indicator of the body’s inflammatory response. Under conditions of immune system stimulation, NF-κB migrates to the nucleus and promotes the release of various pro-inflammatory factors, such as tumor necrosis factor (TNF), interleukin IL-1β, transforming growth factor-β1 (TGF-β1), and anti-inflammatory factors (IL-6) [27]. Among the anti-inflammatory factors released during high-intensity exercise, IL-6 significantly influences fatigue from physical exertion [38].

IL-6 plays a crucial role in regulating the acute phase of inflammation, exhibiting both anti-inflammatory effects during moderate-to-intense physical exercise and pro-inflammatory effects during prolonged, intense physical activity. Research indicates that elevated levels of IL-6 are significant contributors to body fatigue. The signal transducer and activator of transcription 3 (STAT3) is a key mediator of IL-6’s actions and is essential for regulating various pathological processes, including inflammatory and immune responses. Acute exercise poses a significant challenge to whole-body metabolism, and during recovery, metabolic adjustments are required in multiple tissues to restore metabolic homeostasis. Skeletal muscle IL-6 may play an important role in regulating skeletal muscle substrate utilization, basal and exercise-induced adaptations, adipose tissue glucose uptake, and lipolysis during post-exercise recovery [41].

Taken together, this indicates that IL-6 in skeletal muscle promotes restoration of metabolic homeostasis during post-exercise recovery by regulating skeletal muscle metabolism and mitochondrial biogenesis [41]. MKT regulatory activity is due to close interaction via cross-talking with other cytokines. Both plasticity and cytokine crosstalk are significantly associated with the pro- and anti-inflammatory/regenerative properties of IL-6-type cytokines.

### 5.3. JAK-STAT3 Signaling Pathway in the Protective Activity of MKs and MKT

It is important to note that using MKs and MKT during intense physical activity and oxidative stress can reduce the excess of inflammatory cytokines and pro-inflammatory factors, while also promoting the synthesis of chaperones as protective agents. One of the main functions of molecular chaperones is to prevent the aggregation of misfolded proteins, which is why many chaperone proteins are classified as heat shock proteins because the tendency for proteins to aggregate is enhanced by heat and oxidative stress. Other types of chaperones are involved in transport across membranes, such as mitochondrial membranes [25,37].

As shown in Figure 4, the JAK-STAT3 signaling pathway, which is under the control of IL-6, is involved in various biological processes, including proliferation, angiogenesis, survival, immune modulation, and metabolism. Glycoprotein 130 is a transmembrane protein that is a founding member of the toll-like cytokine receptor class. It forms one subunit of the type I toll-like cytokine receptor in the IL-6 receptor family. Janus kinases (JAKs) are a family of intracellular non-receptor tyrosine kinases that transmit cytokine-mediated signals via the JAK-STAT pathway. In addition to its well-known role in transporting signals between the cytoplasm and the nucleus during cytokine signaling, STAT3 has also been identified within mitochondria. The presence of mitochondrial STAT3 is believed to play a significant role in modulating cellular metabolism, primarily through its effects on the respiratory electron transport chain [37,38,41].

Recent evidence has emerged of the non-canonical and non-genomic activity of STAT3: in a non-classical pathway, heterodimeric STAT3 is re-routed to mitochondria to regulate distinct functions. Of particular interest is its ability to regulate the activity of ETC and the opening of the mitochondrial permeability transition pore (MPTP). This activity has broad implications, including cell survival and the production of ROS and ATP in both normal tissues and pathological conditions. Despite these important observations, most of the questions remain unanswered regarding the mechanism of STAT3’s mitochondrial activity [37,38,41,42].

As shown in Figure 4, the regulation of mitochondrial respiration via STAT involves cell protection aimed at reducing cellular damage during oxidative stress and various diseases. Thus, current knowledge about mitochondrial STAT3 as an additional regulator of the ETC, ATP biosynthesis, and ROS sheds considerable light on the likely mechanisms of the protective action of MKT and other sea cucumber MKs on cellular energy processes under oxidative stress [37,41].

It is also necessary to note the important protective role of chaperones. Misfolding, aggregation and accumulation of proteins are toxic elements in the progression of a broad range of diseases. Molecular chaperones enable a cellular defense by reducing or compartmentalizing these insults. Small heat shock proteins like Hsp22 can facilitate their proper folding or refolding, sequestration or clearance of proteins. Hsp22 represents a previously undescribed activator of both nuclear and mitochondrial functions of STAT3, and its deletion in the context of pressure overload in vivo accelerates the transition into cell failure and increases mortality. The gene associated with retinoid-IFN-induced mortality 19 (GRIM-19) is an essential subunit of the mitochondrial respiratory chain complex I. Studies have demonstrated that GRIM-19 targets multiple signaling pathways and plays a critical role in controlling cell death and growth. Overexpression of GRIM-19 induces cell death while its suppression or inactivation promotes cell growth [42,43,44].

As shown in Figure 4, Hsp22 and GRIM-19 as key elements of the mitochondrial STAT3 signaling pathway may play an important role in the above protective activity of MKs and MKT. The primary function of mitochondria is energy production, where processes such as pyruvate oxidation and ATP synthesis occur. Recent research findings have identified mitoSTAT3 as a regulator of mitochondrial function homeostasis through its controlling influence on the electron transport chain (ETC) and Ca^2+^ exchange. Mitochondrial STAT3 may be involved in respiratory supercomplexes to stimulate ETC activity and ATP synthesis without increasing ROS production. It becomes evident that these non-classical functions of STAT3, which sharply increase biosynthesis under the influence of MKT, play an important role in the antioxidant and anti-inflammatory activities of MKs [25,37,42,43,44].

The obtained data suggest that these MKTs and other MKs may have a stimulating effect on oxidative phosphorylation under conditions of oxidative stress, implying their potential successful use in the treatment of various diseases, combating fatigue and accelerating recovery after heavy physical and mental work. Thus, the functions of mitochondrial STAT3 imply the necessity of its regulation for developing new treatment methods for inflammation-related diseases and non-traditional approaches for their control.

## 6. Pleotropic Mechanisms of Protective Action of MKs and MKT in Oxidative Stress and Heavy Physical Exercise

Cellular metabolism encompasses specific biochemical pathways to generate energy and essential biomolecules necessary for cell survival and growth. Strict genetic and proteomic regulation of these pathways occurs by various mechanisms. When environmental conditions change, cells activate multiple regulatory mechanisms to sustain homeostasis and ensure their proper functioning. The above-mentioned points indicate a close relationship between the antioxidant, anti-inflammatory, and adaptogenic activity of MKT and their positive effects on cellular metabolism and energy at the molecular level [34].

**Figure 5:**
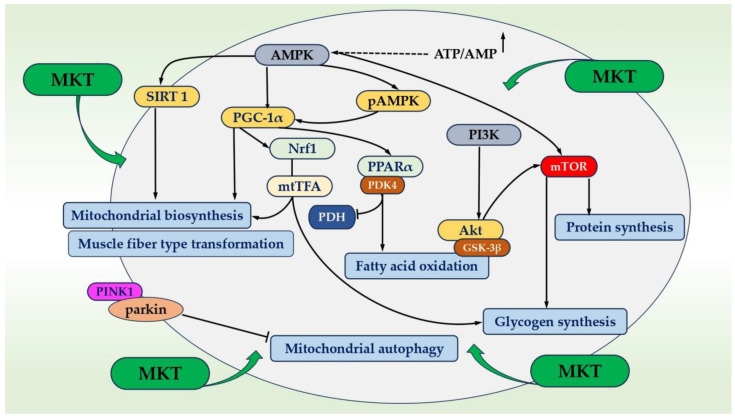
Possible ways of protective action of MKs and MKT in signaling and metabolic pathways under oxidative stress and heavy physical exercise. Adapted from Ref. [34]. Abbreviations: AMP, 5′-adenosine monophosphate; ATP, 5′- adenosine triphosphate; AMPK, 5′-adenosine monophosphate-activated protein kinase; GSK, 3β-glycogen synthase kinase 3β; MKT, matrikines *A. japonicus*; mTOR, the mammalian target of rapamycin; NRF1, nuclear respiratory factor 1; parkin, ubiquitin ligase; PDH, pyruvate dehydrogenase; PDK4, pyruvate dehydrogenase lipoamide kinase isozyme 4; PGC-1α, peroxisome proliferator-activated receptor gamma coactivator 1-alpha; PI3K, phosphoinositide 3-kinase; PINK1, intimately involved with mitochondrial quality control by identifying damaged mitochondria and targeting specific mitochondria for degradation, PPARα, peroxisome proliferator-activated receptor; SIRT1, deacetylates transcription factors that contribute to cellular regulation (reaction to stressors, longevity).

As depicted in Figure 5, metabolic and energetic exchanges are regulated through signaling pathways consisting of AMPK and its associated downstream factors/pathways, which can be modulated by MKs. Prolonged fatigue impedes normal metabolism and the ability to overcome physical exertion. Recovery from physical exertion fatigue involves changes in several cell biochemical pathways, primarily AMPK, PI3K/Akt, NRF2/ARE, NF-κB, PINK1/Parkin, as well as MAPK signaling pathways that are involved in the oxidative stress balance and the switching of muscle fiber type, provide protective effects, and act as energy supply mediators [34].

The protein PDK4, located in the mitochondrial matrix, inhibits the pyruvate dehydrogenase complex by phosphorylating one of its subunits, reducing the conversion of pyruvate, formed from glucose and amino acid oxidation, into acetyl-CoA, and contributes to glucose metabolism regulation [26]. In skeletal muscles, the mammalian target of rapamycin (mTOR) is a key signal that helps coordinate various signaling pathways and nutrient responses. One important pathway involving mTOR is the AMPK/mTOR axis, which helps regulate oxidative stress and enables cells to adapt for survival. Additionally, the PI3K/Akt/mTOR pathway plays a critical role in promoting muscle protein synthesis and contributes to muscle growth, ensuring that muscle cells receive the energy they need [26].

Recent comprehensive research results on the physiological mechanism of fatigue during high physical exertion allow for a systematic analysis of the complex “network” of signaling pathways molecularly associated with fatigue onset, as well as determining the system of interactions among the involved molecules [34].

However, the protective mechanisms of action of MKT, engaged in reducing fatigue during intense physical activity, are highly complex, still insufficiently understood, and require further in-depth research. It is undeniable that the protective and regulatory action of MKs from sea cucumbers, particularly MKT, opens up new possibilities for protecting the body from diseases caused by excessive physical exercises and provides a basis for the prevention and treatment of stress of various etiologies.

It seems that MKs and MKT will take a worthy place in the cohort of this type of protective agent with antioxidant and anti-inflammatory activity. The results presented and discussed here will aid in the development of new health products and provide theoretical and scientific groundwork for future research.

Mitochondrial biogenesis attracts special attention as a potential target for the treatment of diseases that currently have no effective therapeutic agents. Mitochondria, as the main producer of ROS and the main producer of antioxidants, play a decisive role in the processes mediating apoptosis and detoxification in cells and are amenable to pharmacological control. Pathways associated with mitochondrial biogenesis as a compensatory adaptation to energy deficiency during oxidative stress (PGC-1, AMPK, and others) have been described. To trigger AMPK signaling cascades, stilbene resveratrol, quercetin, or other bioflavonoids are used as agonists of Sirt1, also known as NAD-dependent deacetylase sirtuin-1. Other strategies currently in use include adding antioxidant supplements to the diet such as L-carnitine, coenzyme Q10, MitoQ10 and other mitochondria-targeting antioxidants, as well as N-acetylcysteine, a whole vitamin complex, sodium pyruvate or α-lipoic acid, which determine cellular redox homeostasis [45].

It is also important to mention the well-known oxygenated carotenoid astaxanthin (ASX), which has pronounced antioxidant and anti-inflammatory activity, was recently studied in an experimental model of chronic high-intensity interval training (HIIT). Reactive oxygen and nitrogen species are required for exercise-induced molecular adaptations; however, excessive exercise may cause cellular oxidative distress. Compared to the HIIT control mice, the ASX-treated HIIT mice reduced malonaldehyde levels and upregulated the expression of NRF2 forkhead box O3 (FOXO3a). In the group of mice undergoing HIIT with astaxanthin (ASX) treatment, specific genes associated with antioxidant defense and cellular health were found to be upregulated. These include NAD(P)H quinone dehydrogenase 1 and glutamate-cysteine ligase catalytic subunit (GCLC), which are influenced by NRF2, as well as SOD2, and regulated by FOXO3a and glutathione peroxidase 4. Additionally, energy-sensing proteins like AMPK, SIRT1, and SIRT3 also showed increased expression in the ASX-treated group compared to the controls. Moreover, PGC-1α, which is activated by AMPK and SIRT1, was elevated in the ASX-treated HIIT group. This increase in PGC-1α stimulated the expression of key factors involved in mitochondrial function, such as nuclear respiratory factor 1, mitochondrial transcription factor A, and other mitochondrial proteins like isocitrate dehydrogenase NADP(+) 2 and ATP50. Additionally, the ASX treatment resulted in increased levels of mitochondrial fusion factors such as Mfn1, Mfn2, and OPA1. In summary, supplementing with ASX seems to effectively reduce oxidative stress while boosting antioxidant defenses and promoting mitochondrial biogenesis during high-intensity interval training in mice [46].

The protective effect of a mixture of oxygenated carotenoids (ASX, zeaxanthin) from starfish *P. pectinifera* was recorded in experimental testing using various models of inflammation [47].

Thus, pharmacological agents with different structures and antioxidant and anti-inflammatory activity can use a variety of biochemical pathways to exert a stabilizing effect on the redox balance at the cellular and physiological level, which often underlies their adaptogenic activity.

## 7. Plausibility of Adaptogenic Activity of MKs and MKT

ROS and RNS, such as nitric oxide, are known to have both damaging and beneficial roles. These molecules are typically produced by several enzymes, including nitric oxide synthase (NOS) and various isoforms of NAD(P)H oxidase. However, when there is an overproduction of ROS—whether from the mitochondrial electron transport chain or excessive activation of NAD(P)H—it can lead to oxidative stress. This harmful condition can significantly damage various cellular components, including membranes, lipids, DNA and proteins [45].

Recent studies have revealed a wide variety of natural active ingredients with effective anti-fatigue properties, opening up avenues for research in the development of new adaptogenic agents. Inflammation and tissue damage induced by intensive physical exercises are key factors causing fatigue. This is because physical exertion injuries lead to the production of a large amount of highly aggressive ROS radicals and inflammatory factors such as LPS, interleukin-1β (IL-1β), interleukin-6 (IL-6), and tumor necrosis factor-α (TNF-α) [46,47,48,49].

The roles of ROS/RNS in physiological functions have been widely studied, especially regarding how cells adapt to exercise training. However, there are still many debates surrounding the adaptations induced by these reactive species during physical training. Key players in generating superoxide (O_2_^·−^) and hydrogen peroxide (H_2_O_2_) are NADPH oxidase (NOX) and xanthine oxidase (XO), which are also involved in signaling pathways. As a result, they may be crucial for muscle adaptation during exercise. Future research should focus on understanding the upstream signaling processes that activate NOXs and XO in relation to exercise training. Additionally, it is important to further investigate the primary functions of NOXs. The specific mechanisms by which antioxidant genes are finely tuned to maintain redox balance under different exercise conditions also need to be elucidated, particularly regarding the NRF2-Keap1 and NF-κB pathways. Lastly, the biochemical and physiological adaptations of skeletal muscle during and after aerobic exercise are complex and require more in-depth research, particularly concerning the roles of oxidative stress and antioxidants [50].

An increased level of ROS/RNS leads to oxidative stress, which exacerbates muscle inflammation caused by physical exertion, resulting in decreased physical strength. It has been established that elevated levels of pro-inflammatory cytokines activate nuclear factor κ-B (NF-κB), creating a vicious cycle of inflammatory reaction and mitochondrial dysfunction [51]. Damaged mitochondria produce more ROS, falling into a negative feedback loop, resulting in diminished muscle strength and increased fatigue. In the presence of MKTs, a directly opposite protective effect is observed in the form of increased muscle strength and reduced fatigue. It can be hypothesized that MKTs act as protective agents against oxidative stress, exerting their action through the NRF2-dependent pathway, making MKTs promising candidates for the development of peptide adaptogens.

Recent studies on the protective activity of MKTs against oxidative stress and fatigue have attracted significant attention as a potentially beneficial therapeutic adaptogenic agent for human health. MKTs are characterized by relatively high biological activity, low molecular weight, easy absorption, and low toxicity. Excessive production of free radicals is a significant cause of overwork and fatigue, and enhancing antioxidant and anti-inflammatory potential helps eliminate free radicals formed during physical and mental overload, thereby reducing overwork [32].

**Figure 6:**
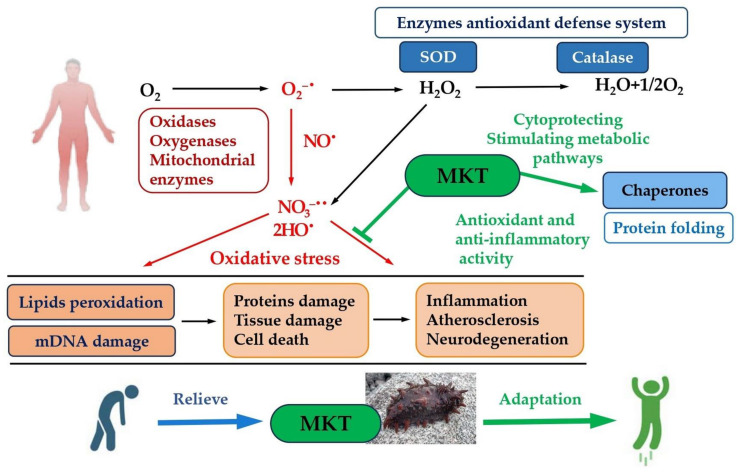
Adaptogenic mechanisms of the protective effect of MKs and MKT on the human body during oxidative stress, which causes fatigue and loss of vitality. Abbreviations: MKT, matrikines *A. japonicus;* SOD, superoxide dismutase.

Figure 6 presents a probable chain of sequential events of the protective adaptogenic action of MKT at the molecular and cellular levels in modeling oxidative stress in experimental animals. This figure illustrates various signaling pathways of the adaptive response of cells to stress, contributing to increased viability. The application of MKT during oxidative stress, heavy physical exercises, and cognitive problems caused by heavy mental work allows the activation of intracellular signaling cascades and transcription factors that induce the expression of protective proteins. Mild cellular stress resulting from increased ROS levels and decreased ATP activates adaptive stress response pathways, including those that activate antioxidant enzymes and chaperone proteins. A characteristic feature of adaptogens is that they act as eustressors (i.e., “good stressors”) [52].

Thus, the pharmacological activity of adaptogens, including MKs and MKT from other sea cucumbers, is nonspecific and is associated with more than one of the receptors affecting key mediators of the adaptive stress response at the intracellular and extracellular levels of communication [32]. Consequently, MKT can effectively regulate and protect the antioxidant system of the body, control ATP levels and energy metabolism, reduce levels of ROS and other pro-inflammatory factors, as well as decrease the content of toxic metabolites in the serum, increase glycogen reserves, contribute to a significant increase in endurance in experimental animals, and efficiently alleviate fatigue, demonstrating all the characteristics/properties of adaptogenic substances. It should be noted that a wide variety of natural active ingredients with adaptogenic effects has been identified in studies of traditional Chinese medicines, opening up broad possibilities for the search for new effective adaptogens, including MKs [48,49,51,52].

To counteract stressors, cells employ quality control measures involving chaperones that help preserve and regulate both cellular and mitochondrial functions. Recent proteomic studies have begun to identify the specific clients of certain chaperones, highlighting many proteins that are essential for the proper organization of the tricarboxylic acid (TCA) cycle and oxidative phosphorylation (OXPHOS) assemblies. Chaperones play a critical role in enhancing the efficiency of metabolic processes by facilitating the formation of metabolons—complexes where enzymes work together—and ensuring effective substrate channeling among the enzymes within supramolecular protein assemblies. Moreover, chaperones aid in the recruitment and trafficking of targeted proteins for degradation, which helps redirect cellular resources to other metabolic pathways as needed. However, the investigation of these processes in the context of metabolic stress is still in its infancy and requires further research to ascertain the extent of regulation. It also remains to be determined whether this regulation can be specific or selective for various metabolic enzymes. Understanding these dynamics could provide insights into how cells maintain metabolic flexibility and adapt to changing conditions [53].

**Figure 7:**
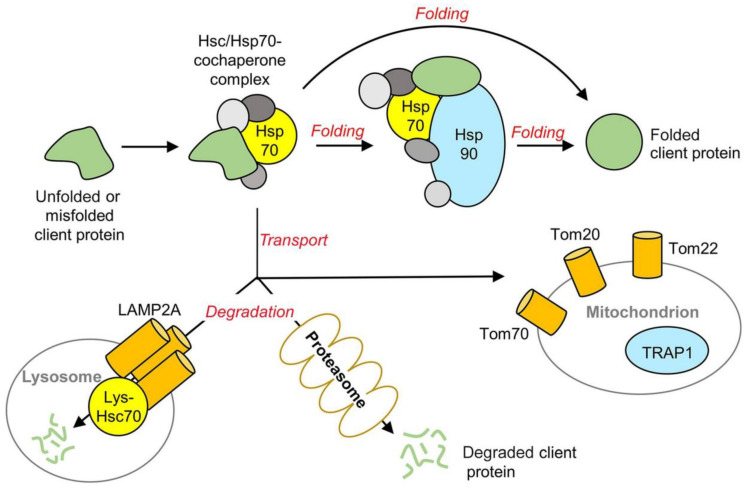
A generalized scheme for Hsp70 and Hsp90 chaperone action on protein clients. Adapted from Ref. [53]. Abbreviations: Hsc70, heat shock cognate 71 kDa; Hsp (Hsp70 and Hsp90), chaperones (heat shock proteins); LAMP2A, lysosome-associated membrane protein type 2A; Tom (Tom20, Tom22, and Tom70), translocases of the outer mitochondrial membrane; TRAP1, tumor necrosis factor receptor-associated protein 1.

Extensive biochemical and structural studies have been performed to better understand chaperones, in particular Hsp70 and Hsp90, in adaptogenic activity. The results of these studies are presented in Figure 7.

Unfolded or misfolded proteins that arise under stress conditions are recruited into Hsp70 complexes composed of co-chaperones such as those in the DnaJ and BAG (gray) families [53]. This sequestration (or stabilization) allows the client protein to be transported either to Hsp90 for folding, to mitochondrial translocases (Tom20, Tom22, Tom70) for importing, or to the machinery (LAMP2A receptors on lysosomes, proteasome) responsible for degradation. Current models describing the mechanism of action of Hsp70 also suggest that it may fold proteins in the absence of Hsp90 or promote the unfolding of misfolded proteins and aggregates [53].

How do chaperones decide which processes to regulate? Given the diverse array of client proteins they interact with, there must be specific biochemical or structural characteristics that facilitate the recruitment of certain clients over others. These characteristics can help clarify how chaperones act like switches, capable of activating one pathway while inhibiting another. Two main factors that could influence this selectivity are the types of co-chaperones involved and the state of post-translational modifications on the chaperones themselves. While various molecular chaperones may function redundantly, the presence of such a wide variety of client proteins indicates that there is also significant diversity among these chaperones. Furthermore, chaperones are quite sensitive to modifications after translation, which can alter their function. Understanding the intricate network of these post-translational modifications, often referred to as the “chaperone code”, and their relationship to specific chaperoning roles and client recruitment offers a substantial challenge but also exciting opportunities for developing new research tools and methodologies [53].

Meanwhile, the question of what the molecular mechanisms of the interactions of matrikines are, in particular MKs and MKT, with various chaperones that lead to a protective adaptogenic effect remains open for further research. Considering the above, there is every reason to believe that MKT could become the crown jewel among protective adaptogenic substances widely used in modern practice as important elements of healthy nutrition and supplementary therapy: maintaining health (based on homeostasis, adaptation, and other protective mechanisms of the body), increasing health reserves and, as a result, improving the quality and duration of life.

## 8. Conclusions

In recent years, original enzymatic technologies have been developed to obtain structurally diverse and biologically active marine-derived compounds (MKs) from various species of sea cucumbers. Some of these substances can serve as valuable raw materials for the production of functional food products and pharmaceuticals. This review analyzes numerous publications, mainly related to MKs from sea cucumbers, which possess antioxidant, anti-inflammatory, and adaptogenic properties. These MKs are non-toxic, safe for long-term use, and exhibit high antioxidant and anti-inflammatory protective effects in cellular and animal models, which are widely used in modern pharmacology. Currently, many countries around the world are conducting extensive scientific research to “decode” the molecular mechanisms of the antioxidant, anti-inflammatory, and adaptogenic actions of active components from Eastern medicine recipes. In this context, MKs from various sea cucumbers are actively being studied. The analysis of publications conducted in this review shows that MKs from sea cucumbers act on molecular and cellular levels through various mechanisms, increasing the body’s resilience to adverse stressors.

It has been demonstrated that the administration of MKs during stress, illness, and/or heavy physical exertion leads to a reorganization of biochemical and signaling processes, activating antioxidant protection and cellular energy supply: stimulation of lipid metabolism, reduction in ATP consumption, and increased synthesis in mitochondria and glycogen in muscles, optimization of intracellular amino acid formation, and their transport from outside. Notably, the increase in performance induced by MK administration is not accompanied by subsequent negative side effects typically associated with various doping agents.

This review presents a probable chain of sequential events of the protective action of MKs and MKT at the molecular level in modeling oxidative stress on cells and experimental animals. The logic behind the development and application of therapeutic agents based on sea cucumber-derived MKs is clear and fully aligned with the logic of the modern standard pharmacological approach—producing specific compounds to interact with a specific target. Nevertheless, aggregated results of contemporary research indicate the “pleiotropic” multiple nature of the antioxidant, anti-inflammatory and adaptogenic effects of sea cucumber-derived MKs. Despite the accumulation of important information about the mechanism of action of sea cucumber-derived MKs, future research should focus on assessing their pharmacokinetic and pharmacodynamic properties, as well as identifying their molecular targets to enhance their effectiveness in combating stress and treating various inflammation- and stress-related diseases. Following preclinical trials, these MKs could be recommended for use as the basis for developing new dietary, therapeutic, and preventive agents, as well as components of functional food products that improve health and increase human lifespan.

Furthermore, the search and systematic research of the biological activity of various MKs based on their structural characteristics should be continued. Ultimately, this may lead, on the one hand, to the creation of new promising therapeutic agents and, on the other hand, to their widespread use as elements of functional nutrition among different populations. The active utilization of sea cucumber-derived MKs for medical purposes may accelerate the implementation of new technological schemes for their comprehensive processing and stimulate the development of aquaculture, thus promoting the rational use of these marine bioresources.

## Figures and Tables

**Table 1:** Anti-inflammatory action of MKs from sea Echinodermata in acute inflammation model [8].

Group	Number of Animals	Dose (mg/kg)	Paw Weight 5 h after the Inflammation Induction (×10^−2^ g)	Inhibition, %
Control	4	-	81.25 ± 1.70	-
Indomethacin	4	10	39.02 ± 2.08	52.0 ± 4.06 *
MKC	4	10	43.75 ± 1.0	46.15 ± 2.09 *
MKC	4	20	55.25 ± 5.01	32.04 ± 6.06 *
MKT	4	10	48.78 ± 3.20	40.0 ± 4.09 *
MKT	4	20	53.25 ± 1.25	34.46 ± 2.47 *
MKUm	4	10	46.25 ± 4.08	43.08 ± 5.26 *
MKUm	4	20	47.25 ± 0.06	41.85 ± 1.02 *
MKUn	4	10	53.03 ± 1.19	34.77 ± 2.32 *
MKUn	4	20	35.75 ± 1.11	56.04 ± 2.87 *
MKS	5	10	34.29 ± 4.10	43.02 ± 2.47 *
MKS	5	20	37.32 ± 3.24	45.17 ± 5.20 *

Abbreviations: matrikines: *A. japonicus* (MKT); sea star *P. pectinifera* (MKS); cucumaria *C. japonica* (MKC); trepang *A. japonicus* (MKT); sea urchins *Sc. mirabilis* (MKUm) and *St. nudus* (MKUn). CD-1 mice (20 ± 2 g) were used to model acute inflammation. The results are presented as m ± SD (standard deviation) from 3 independent experiments. * The differences are significant at *p* < 0.05.

## Data Availability

Not applicable.
